# Editorial: Perspectives in extra-intestinal microbiome 2025

**DOI:** 10.3389/fcimb.2026.1824939

**Published:** 2026-04-10

**Authors:** Shi Huang, Xin Xu

**Affiliations:** 1 Faculty of Dentistry, The University of Hong Kong, Hong Kong, Hong Kong SAR, China; 2 The State Key Laboratory of Oral Diseases and National Clinical Research Center for Oral Disease, West China Hospital of Stomatology, Sichuan University, Chengdu, China

**Keywords:** extra-intestinal microbiome, microbial dysbiosis, microbiome-targeted therapies, oral microbiome, skin microbiome, urinary microbiome

## Introduction

The human microbiome has emerged as a critical determinant of health and disease, extending far beyond its traditional gastrointestinal confines. While the gut microbiome remains the most extensively studied ecosystem, the past decade has witnessed a paradigm shift toward understanding microbiomes at extra-intestinal sites—distinct communities that interact with host physiology through specialized niche adaptations. This Research Topic brings together five contributions that illuminate recent advances across diverse anatomical locations, methodological innovations, and therapeutic frontiers in extra-intestinal microbiome research.

## Advancing methodological rigor and integration

A persistent challenge in microbiome science is the heterogeneity of findings across studies, often stemming from methodological variability. Han and Ding address this critical issue in the context of periodontitis research, advocating for standardized protocols in study design, sampling, and analysis. Their perspective highlights how geographic bias, inconsistent age stratification, and varying definitions of disease states have complicated cross-study comparisons. Importantly, they argue for moving beyond compositional profiling toward functional-driven analyses using metatranscriptomics and emerging single-cell technologies. This call for methodological harmonization resonates across all extra-intestinal microbiome fields, where low-biomass samples and contamination risks pose unique challenges.

## The urinary microbiome: from sterility dogma to clinical translation

Perhaps no extra-intestinal site exemplifies the overturning of medical dogma more dramatically than the urinary tract. Zhang et al. provide a comprehensive review of the urinary microbiome’s role in non-infectious urological diseases, demonstrating how 16S rRNA sequencing and metagenomics have revealed diverse microbial communities even in healthy individuals. Their synthesis reveals disease-specific signatures: enrichment of *Fusobacterium* and *Acinetobacter* in bladder cancer, *Propionibacterium* elevation in prostate cancer, and *Lactobacillus* depletion across multiple pathologies. Particularly noteworthy is the emerging potential for microbiome-based diagnostics—machine learning models achieving >80% accuracy for cancer detection—and the prospect of microbiome-targeted therapies, including engineered bacteria for kidney stone prevention. This work underscores how urinary microbiome research has matured from descriptive cataloging toward mechanistic understanding and clinical application.

## Reproductive and perinatal health: microbial dynamics across the maternal-infant axis

Xie et al. examine the pregnancy microbiome’s dynamic reconfiguration across vaginal, gut, and oral compartments, and its profound implications for preterm birth (PTB). During healthy pregnancy, the vaginal microbiome stabilizes toward *Lactobacillus* dominance, while gut communities shift toward reduced diversity and pro-inflammatory profiles. Dysbiosis—characterized by diminished *Lactobacillus* and anaerobic expansion—emerges as a consistent risk factor for PTB through multiple pathways: local immune dysregulation, systemic inflammation, and microbial translocation. The authors highlight promising probiotic interventions, though they appropriately caution that strain-specific efficacy and optimal timing require further validation. This contribution exemplifies how extra-intestinal microbiome research must account for hormonal, immunological, and metabolic co-variation across physiological states.

## The oral-cardiovascular axis: systemic consequences of local dysbiosis

Wu et al. articulate the multidimensional relationships between oral microbiota and cardiovascular disease (CVD), moving beyond simple correlation to elucidate plausible mechanisms. Periodontal pathogens including *Porphyromonas gingivalis* and *Fusobacterium nucleatum* contribute to hypertension, atherosclerosis, myocardial infarction, and endocarditis through diverse pathways: bacteremia-induced endothelial dysfunction, trimethylamine N-oxide (TMAO) metabolism, nitric oxide dysregulation, and platelet activation. The oral cavity thus serves as both a diagnostic window and therapeutic target for systemic disease—a concept with significant preventive implications given the global burden of CVD. Their review importantly identifies gaps requiring longitudinal studies with standardized baselines and controlled interventions.

## cosy Cutaneous estems: restoring balance in atopic dermatitis

Zhong et al. present a nuanced view of skin microbiome dysbiosis in atopic dermatitis (AD), framing disease pathogenesis as a self-reinforcing cycle involving barrier dysfunction, immune dysregulation, and microbial imbalance. *Staphylococcus aureus* dominance emerges as a central node in this pathological network, with the loss of commensal anaerobes and reduced microbial-derived metabolites (short-chain fatty acids, indole derivatives) compromising colonization resistance. The authors survey emerging bacteriotherapeutic strategies—from *Roseomonas mucosa* and *Staphylococcus hominis* transplantation to precision phage therapy—while emphasizing that successful translation requires strain-level functional characterization and personalized approaches based on multi-omics stratification.

## Synthesis and future directions

Collectively, these contributions reveal several convergent themes in extra-intestinal microbiome research. First, technological advancement continues to reshape our understanding of microbial diversity at body sites previously considered sterile or homogeneous. Second, disease associations increasingly yield to mechanistic insights linking specific taxa or functional pathways to host pathophysiology. Third, therapeutic translation—while promising—demands rigorous standardization and personalized frameworks to account for inter-individual variation.

Looking forward, several priorities emerge. Multi-omics integration (combining metagenomics, metatranscriptomics, metabolomics, and host transcriptomics) will be essential for moving beyond correlation to causation. Longitudinal cohort studies with standardized protocols, as advocated by Han and Ding, will clarify temporal dynamics and enable predictive modeling. Finally, the development of live biotherapeutic products and targeted microbial interventions requires navigable regulatory pathways and robust safety profiles.

The extra-intestinal microbiome represents a new frontier in precision medicine—one where ecological restoration may complement or supersede traditional pharmacological approaches. We hope this Research Topic stimulates continued interdisciplinary collaboration and accelerates the translation of microbiome science into clinical practice.

